# Paraplegia Following Ultrasound-Guided Caudal Epidural Block in Chronic Lumbosciatica: What Can Be Learned From This Complication?

**DOI:** 10.7759/cureus.48916

**Published:** 2023-11-16

**Authors:** André Teixeira, Jorge Barbosa

**Affiliations:** 1 Physical Medicine and Rehabilitation, Centro Hospitalar Universitário de Lisboa Central, Lisbon, PRT; 2 Physical Medicine and Rehabilitation, Centro Hospitalar de Lisboa Ocidental, Lisbon, PRT

**Keywords:** low back pain, radiculopathies, caudal epidural block, caudal anesthesia, ultrasonography, flaccid paraplegia

## Abstract

Caudal epidural block is a procedure that involves the injection of anesthetic agents through the sacral hiatus, commonly used for regional anesthesia. It is also valuable for chronic pain management in lumbosacral conditions, trauma, and palliative care. Ultrasound-guided caudal epidural blocks can be an alternative to fluoroscopy-guided techniques and have demonstrated a notably high success rate. However, despite both techniques being generally regarded as safe, they can lead to severe complications, such as abscesses, epidural hematomas, and subdural punctures. Furthermore, documented instances of lumbosacral region anomalies, stemming from either anatomical variations or underlying pathology, have been associated with an elevated risk of some of these complications. The authors report a rare case of paraplegia following an ultrasound-guided caudal block in a patient with refractory chronic lumbosciatica. This case underscores the need for vigilance in risk assessment and detailed procedural planning. It also highlights the importance of transparent communication, particularly during informed consent, to convey risks and benefits to the patient and their family.

## Introduction

Caudal epidural block is a procedure involving epidural infiltration of anesthetic agents via the sacral hiatus. Various techniques have been described, including blind, fluoroscopy-guided, and ultrasound-guided approaches. It can be used for both diagnostic and therapeutic purposes. The main indications in adults include regional anesthesia for pelvic, abdominal, and lower limb surgeries, pain control in cases of lumbosacral radiculopathy or lumbar stenosis, pelvic pain, trauma-related pain, post-surgical pain, and palliative care [1-3]. The ultrasound-guided technique has a reported success rate of approximately 75% [4]. Despite being considered safe, potential adverse effects may include headaches, nausea, vomiting, vasovagal syncope, sensory or motor function loss, vesico-sphincter disorders, or localized pain. The most frequent severe complications are abscess, epidural hematoma, or subdural puncture [2]. The presence of underlying diseases such as perineural cysts or posterior sacral meningocele, along with anatomical variations, may be correlated with increased susceptibility to developing some of these complications or a failed technique [4,5]. The authors report a rare case of paraplegia following an ultrasound-guided caudal epidural block in a patient with refractory chronic lumbosciatica.

## Case presentation

**Figure 1:**
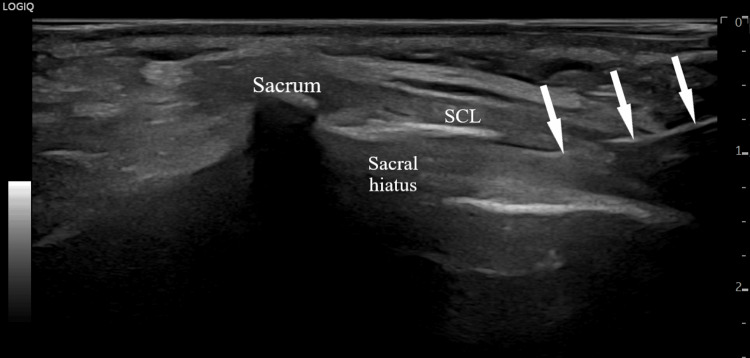
Ultrasound-guided caudal epidural block (sagittal plane). SCL: sacrococcygeal ligament. Arrows indicate needle.

**Figure 2:**
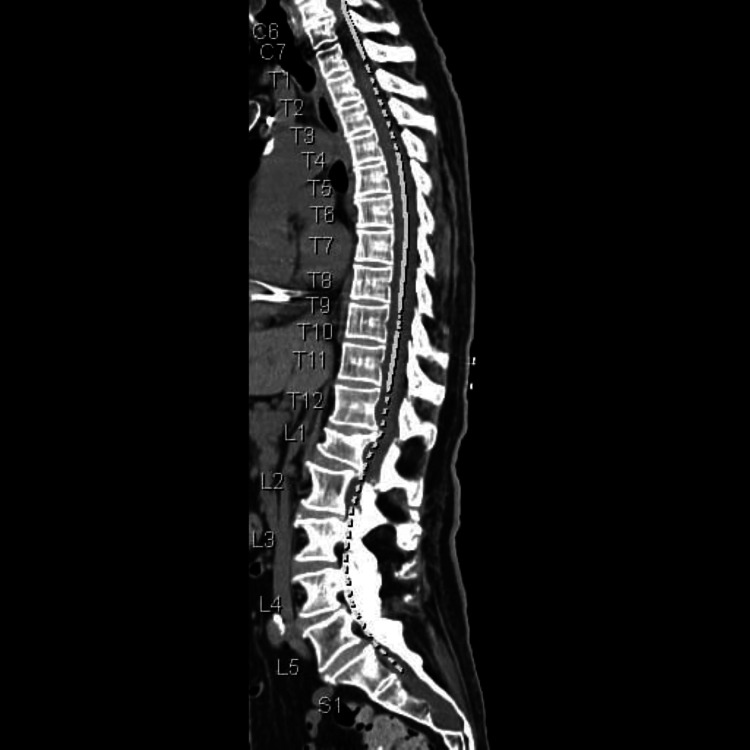
Computerized tomography scan of the spine (sagittal plane).

A 72-year-old woman with a medical history of hypertension and a pacemaker was referred for Physical and Rehabilitation Medicine ultrasound-guided technique consultation due to chronic low back pain of lumbar stenosis affecting L2-L5, bilateral L5-S1 radiculopathy, and bilateral L4-S1 facet arthropathy. Upon examination, she exhibited tenderness with radiating pain to the buttock and positive Kemp test findings with localized pain to the facets of L4-L5 and L5-S1 bilaterally, as well as bilateral positive Lasègue sign with radiating pain resembling an electric shock sensation extending to the dorsum of the feet. From the previous exams performed, there was a lumbar spine computerized tomography (CT) scan taken three years ago, highlighting lumbar stenosis at L2-L5; discopathy at L2-S1; probable bilateral L5 and S1 radiculopathy, and Tarlov cysts. Despite the aging nature of the CT, the difficulties in obtaining a timely appointment, and the need to provide effective treatment for the patient's current symptoms, the proposal to conduct an ultrasound-guided infiltrative procedure was made. After obtaining signed informed consent, bilateral facet joint injections at L4-L5 and L5-S1 were performed using ultrasound-guided technique, with each site receiving 2 cc of 2% lidocaine and 0.5 cc of 4 mg/ml dexamethasone. In the same medical appointment, a caudal epidural block was subsequently administered with 20 cc of 2.5 mg/ml levobupivacaine, 9 cc of 0.9% NaCl, and 1 cc of 4 mg/ml dexamethasone, also under ultrasound guidance (Figure 1). Immediately following the procedure, the patient experienced a sudden onset of paraplegia (grade 0 in all segments of the lower extremities according to the Medical Research Council muscle scale) and hypoesthesia from the T4 to T12 dermatomal level, with bilateral anesthesia distal to T12, without any other symptoms, remaining hemodynamically stable and eupneic. The patient continued to be closely monitored within the Physical and Rehabilitation Medicine department. Due to ongoing complaints and the onset of abdominal pain and urinary retention, she was admitted to the emergency department five hours after experiencing symptoms. An indwelling catheter was inserted, which drained 1,000 cc of clear urine. The lumbar spine CT scan (Figure 2) revealed no acute intracanal collections and showed no evidence of disease progression when compared to the previous CT scan. This prompted a recommendation for magnetic resonance imaging (MRI) for further characterization. However, the MRI was not performed due to the patient's incompatible pacemaker. Regarding motor function and sensory, she demonstrated progressive improvement and complete recovery 12 hours after symptom onset. She was discharged with preserved muscle strength (grade 5 in all segments of the lower extremities according to the Medical Research Council muscle scale) and normal sensory examination in all segments, the ability for spontaneous micturition, and recovery of her regular gait.

## Discussion

**Figure 3:**
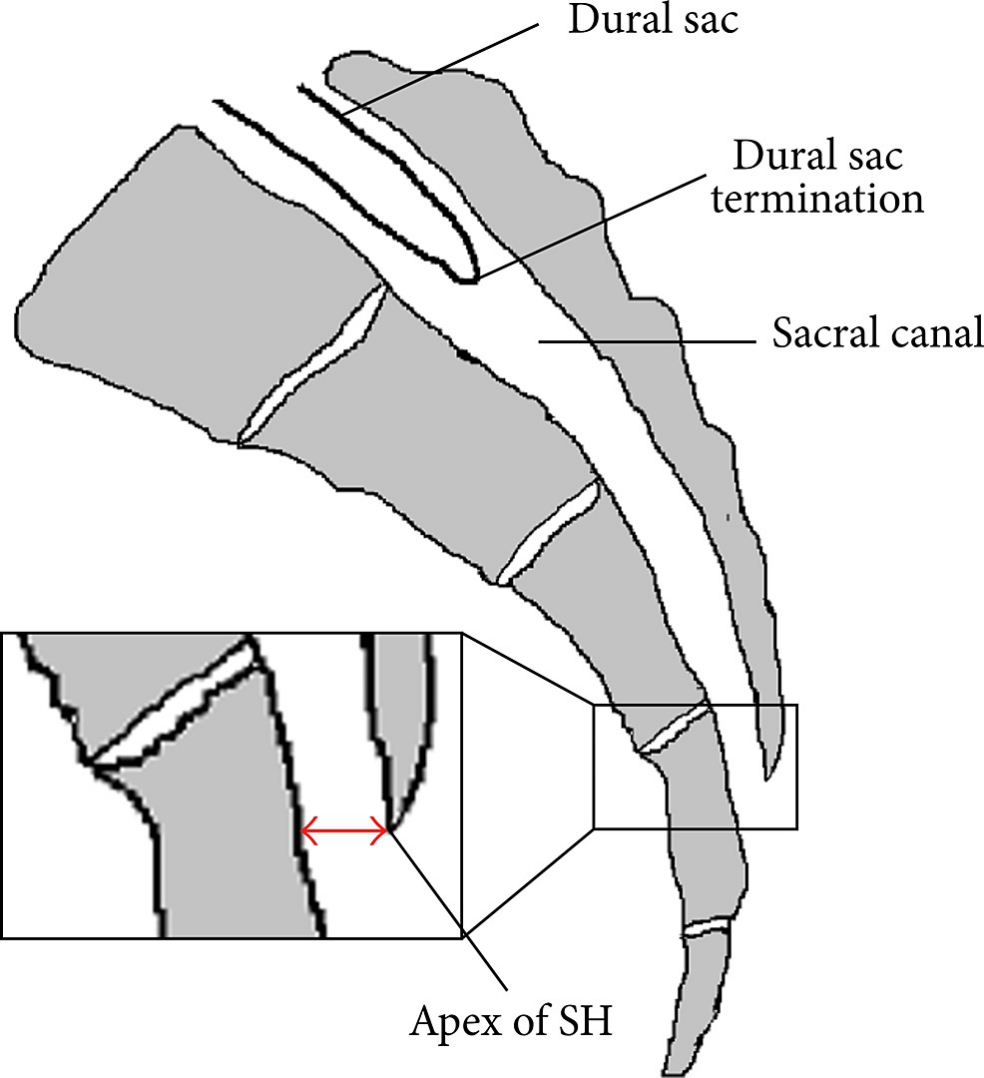
Sagittal view of sacrum and dural sac termination. SH: sacral hiatus. Originally published in Kao and Lin [4]. Used with copyright permission from the original publisher.

Bilateral facet joint injections were performed due to suspected facetogenic pain. This decision was based on the patient's history of progressive pain, radiating pain to the buttock, paraspinal tenderness, and a positive Kemp test, which replicates facet joint loading. It is crucial to emphasize that this procedure serves both therapeutic and diagnostic purposes. It can be considered as the initial step in determining the appropriate treatment for facetogenic pain, which typically involves radiofrequency denervation of the medial branch nerves [6,7]. In light of the clear association with a chronic radicular pain syndrome, as evidenced by the presence of neuropathic pain and a positive bilateral Lasègue sign, the patient underwent a caudal epidural block. In the vast majority of adult cases, the dural sac terminates at the level of S1-S2 (Figure 3). Anatomic variations in the lumbosacral transition have been described, with a prevalence of up to 10% (at the S3 level and, in very rare cases, at the S4 and S5 levels)[4]. Joo et al. also studied the prevalence of these anatomical variations in 2,669 Korean individuals and found that approximately 1.6% of them had anatomical variations or conditions that increased the risk of dural sac puncture [5]. This author reported two cases of accidental dural sac puncture in the context of these anatomical variations [8]. Other similar cases have been described in the literature, albeit rarely, reinforcing the relationship between the presence of these variations and the risk of dural sac puncture [9,10]. In such instances, it may be advisable to perform preprocedural MRI to characterize anatomical structures, including the termination of the dural sac and or other conditions to minimize this risk [11]. The leading hypotheses for the described neurological condition include subdural blockage related to the accidental dural sac puncture or intrathecal blockage, the latter potentially induced by puncturing a perineural cyst (Tarlov cyst). These cysts have a high incidence (4%-9%) in asymptomatic patients and can communicate with the subarachnoid space, allowing for a more proximal dispersion of the anesthetic, thus justifying the presented clinical symptoms [3]. As described in the literature, these are the most common conditions in which dense sensorimotor blocks can occur even with ultrasound-guided low concentration local anesthetic administration. First, it can occur when the dural sac terminates abnormally low, with the dural sac tip located at the lower sacrum. Second, it can occur when the cystic structure is extended to the lower sacrum due to pathological conditions such as sacral meningocele or perineural cyst as the Tarlov cysts [9]. The fact that this patient cannot undergo an MRI due to the presence of a non-MRI-compatible pacemaker prevented the definitive diagnosis. Nevertheless, considering the clinical presentation and the exclusion of other conditions through CT, it is highly likely that one of the two previously described hypotheses was the cause of the neurological condition. The description of Tarlov cysts in a previous CT raises our suspicion in this hypothesis, although there was no information about the location of the cysts, if there was communication with the subarachnoid space and if there was an actual puncture of a cyst during the procedure. The concentration and volume employed for the caudal epidural block, although not explaining the neurological deficits, might contribute to the observed elevated neurological level, despite the literature documenting the safe use of higher doses, such as 20 ml of 0.5% bupivacaine [12]. While the fluoroscopy-guided technique remains the gold standard, the ultrasound-guided approach has gained popularity due to its ease of execution, cost-effectiveness, and reduced risk of puncturing vascular structures, facilitated by Doppler guidance [13]. To prevent this complication in the future, in addition to considering alternative diagnostic methods, such as an initial MRI, adjusting both the concentration and injected volume may help reduce the extent of spread and potentially lower the block level. Moreover, initiating the procedure with a small test dose and a waiting period may be recommended.

## Conclusions

Caudal epidural block, performed under ultrasound guidance, is a secure and widely practiced technique in chronic pain management. It has gained prominence, particularly in cases involving individuals experiencing refractory chronic low back pain with radiculopathy or lumbar stenosis. However, there is always a low risk of potential adverse effects. The neurological condition we describe, although severe, was transient, with the most likely etiology being unintentional subdural or intrathecal blockage. This case highlights the importance of remaining vigilant regarding the risks associated with this procedure and emphasizes the significance of meticulous procedural planning. It also underscores the importance of transparent and comprehensive communication regarding both the risks and benefits of these procedures, especially to the patient and family when obtaining informed consent.
